# Accurate (n,m) Assignment of Single‐Walled Carbon Nanotubes From Electron Diffraction Patterns Via Local Candidate Search and Self‐Consistency Correction

**DOI:** 10.1002/smtd.71039

**Published:** 2026-09-16

**Authors:** Hirotaka Inoue, Hua Jiang, Rui Iwasaki, Anastasios Karakassides, Toshihiko Fujimori, Esko I. Kauppinen

**Affiliations:** ^1^ Department of Applied Physics School of Science Aalto University Espoo Finland; ^2^ Sumitomo Electric Industries, Ltd. Osaka Japan; ^3^ Institute of Applied Physics University of Tsukuba Tsukuba Japan

**Keywords:** chirality, nanobeam electron diffraction, single‐walled carbon nanotube, transmission electron microscopy

## Abstract

The chirality (*n*,*m*) of single‐walled carbon nanotubes (SWCNTs) critically governs their properties and growth behavior, yet reliable assignment from nanobeam electron diffraction (NBED) remains challenging because of analyst‐dependent procedures, manual measurement errors, and nanotube tilt relative to the incident electron beam. Here, a practical and interpretable chirality‐assignment protocol is developed by extending a calibration‐free diffraction‐analysis framework with local candidate search and self‐consistency‐based cross‐check correction. Feature extraction and candidate screening are semiautomated using an ImageJ script and an Excel VBA macro to improve efficiency and reproducibility. The protocol is evaluated using 2394 simulated NBED images spanning diameters of 0.48–2.3 nm, chiral angles of 0°–30°, and tilt angles of 0.5°–16.5°. The exact assignment accuracy increases from 71.6% with conventional TRUNC‐based analysis to 97.7% with the proposed protocol, while errors in the mean diameter, mean chiral angle, and metallic SWCNT fraction are substantially reduced. The improvement is mainly enabled by the newly introduced overlap ratio, which serves as the primary criterion for final candidate ranking. This error‐resilient workflow provides a reproducible route for extracting reliable structural information from experimentally imperfect diffraction patterns without requiring instrument calibration or data‐driven model training.

## Graphical abstract

A practical workflow combining local candidate search, overlap‐ratio ranking, and self‐consistency correction enables reliable chirality assignment of single‐walled carbon nanotubes (SWCNTs) from nanobeam electron diffraction. Semiautomated analysis increases exact (*n*,*m*) assignment accuracy from 71.6% to 97.7% across 2394 simulated patterns, while supporting more reproducible evaluation of SWCNT structural distributions without data‐driven model training or instrument calibration.

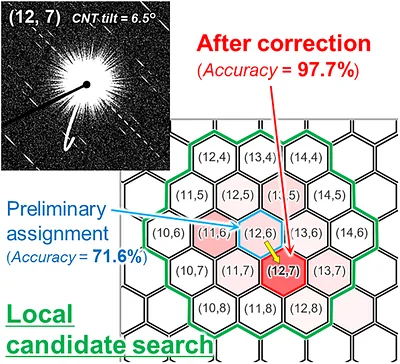

## 1 Introduction

Single‐walled carbon nanotubes (SWCNTs) [1] are known to exhibit markedly different electrical properties [2, 3, 4], as well as growth‐related kinetic and thermodynamic behavior [5, 6, 7, 8], depending on their chirality, which is denoted by the chiral indices (*n*,*m*). The properties and behavior of both individual CNTs and bulk‐scale assemblies are also strongly influenced by the CNT diameter [9, 10] and chiral angle [11], both of which are uniquely determined by chirality. Accordingly, chirality‐selective synthesis has long been recognized as one of the most important targets in the field, alongside the controlled synthesis of CNT length [12, 13, 14], crystallinity [15, 16], and substitutional doping [17, 18, 19], particularly in the context of materials development for practical implementation and device applications of SWCNTs.

In recent years, this field has seen a series of advances in the preferential growth of specific (*n*,*m*) species through improved catalyst design and growth‐field control [6, 8, 20, 21, 22, 23, 24, 25, 26, 27, 28]. For example, in substrate‐based chemical vapor deposition (CVD), highly selective chirality‐controlled growth has been experimentally achieved by using thermally stable W_6_Co_7_ catalysts formed by alloying cobalt and tungsten in combination with optimization of other growth parameters, yielding 95.5% of the metallic chirality (12,6) [24] and 97.4% of the semiconducting chirality (14,4) [29]. Selective synthesis of specific chirality structures has also been explored in floating catalyst chemical vapor deposition (FC‐CVD), which is considered advantageous for industrial processes because it enables continuous production of highly crystalline CNTs under high‐temperature conditions. Chiang et al. reported floating continuous synthesis of CNTs enriched to more than 90% semiconducting species by employing ferrocene and nickelocene as catalyst precursors in FC‐CVD [30, 31]. Inoue et al. reported near‐armchair‐selective growth with strong enrichment toward high chiral angles by employing a mist‐based precursor delivery method and co‐feeding ammonium metatungstate, a tungsten precursor, together with ferrocene [8]. These advances demonstrate that chirality‐selective synthesis has steadily progressed and has the potential to greatly expand the design freedom of SWCNT functionalities.

On the other hand, to firmly establish these advances in synthesis and sustain their development in both academic and industrial contexts, improvements in the accuracy and reproducibility of chirality evaluation are indispensable in addition to advances in synthesis itself. In practice, several methods are available for chirality assignment of SWCNTs, including Raman spectroscopy [32], optical absorption spectroscopy [33], photoluminescence (PL) mapping [34], nanobeam electron diffraction (NBED) using transmission electron microscopy (TEM) [14, 35, 36, 37], and ultrahigh‐resolution TEM image analysis [38, 39]. However, because these methods differ in measurement principle, underlying assumptions, and applicability limits, it has been pointed out that establishing a unified and comparable level of reliability across different studies is not straightforward [40, 41]. Moreover, as chirality‐selective synthesis becomes increasingly sophisticated, the acquisition of large volumes of evaluation data for optimization of synthesis conditions and statistical validation becomes necessary, making human labor a practical bottleneck. Therefore, there is a growing need for analysis protocols that can provide high accuracy and reproducibility while remaining feasible for routine operation at realistic labor cost.

Among the available approaches for chirality assignment of SWCNTs, TEM‐NBED‐based methods have developed as particularly powerful tools because they provide direct access to the atomic structural information of individual SWCNTs. In 1993, Iijima and Ichihashi reported single‐walled tubes with diameters of approximately 1 nm and discussed their structural characteristics based on electron diffraction data [1]. This report represents an important starting point in this field. Thereafter, studies using NBED to determine and map the diameters and helicities of large numbers of SWCNTs extended the practical applicability of NBED‐based (*n*,*m*) assignment [36, 42, 43, 44, 45]. In this context, Jiang et al. introduced dimensionless intrinsic layer‐line spacing and proposed a calibration‐free (*n*,*m*) determination method capable of handling CNT tilt relative to the incident electron beam [37]. While previous NBED‐based methods often required assumptions of normal incidence or relied on laborious trial‐and‐error simulations, this approach simultaneously evaluates the CNT tilt angle and compensates for its effect in the (*n*,*m*) determination. By enabling direct, calibration‐free, and cross‐checkable structural assignment under realistic experimental conditions, this framework significantly improved the practical feasibility of NBED‐based SWCNT chirality determination.

Despite its high potential accuracy, however, NBED‐based (*n*,*m*) assignment still involves analyst‐dependent steps in practical operation, such as layer‐line selection, distance measurement, tilt correction, and determination of integer solutions, allowing manual measurement errors to accumulate and degrade assignment accuracy. Furthermore, especially for CNTs with large diameters [45, 46] or small chiral angles, that is, those close to the zigzag structure [35], manually measured combinations of spot spacings and layer‐line spacings become increasingly similar to those of neighboring chiralities, and the presence of tilt causes these combinations to begin overlapping [35, 36, 37, 46]. As a result, exact identification of the perfectly matching chirality becomes extremely difficult. In other words, even when unique determination is possible in principle, ensuring robustness and reproducibility of the overall analysis workflow requires a strategy that cross‐checks candidate consistency using multiple measured quantities and computational pathways, and that progressively narrows candidates while explicitly accounting for the presence of measurement errors.

In parallel, the introduction of artificial intelligence (AI) and materials informatics (MI) into materials characterization has accelerated in recent years, and studies aimed at automated interpretation and high‐throughput processing of diffraction patterns and microscopy images have rapidly expanded [39, 47, 48, 49]. This trend has also reached CNT chirality analysis from electron diffraction patterns. Recently, Iwasaki et al. demonstrated an AI‐based chirality classification method using a Vision Transformer (ViT) trained entirely on simulated diffraction patterns, without using experimental images for training, and achieved 79.8% exact agreement and 99.3% near‐neighbor agreement with manual assignments for experimental TEM diffraction patterns [50]. This result highlights the potential of simulation‐trained AI models for high‐throughput CNT characterization, while also emphasizing that exact discrimination among neighboring chiralities and the reliability of reference assignments remain critical issues. Although these approaches offer substantial advantages for high‐throughput analysis, practical deployment still faces challenges such as auditability of the output, disentangling error sources, and stability under out‐of‐distribution conditions. In many cases, it remains difficult to make final decisions solely on the basis of black‐box predictions. Therefore, even when incorporating new analytical technologies including AI, the importance of high‐accuracy, physics‐based assignment frameworks that can provide reliable reference data, quantify error sources, and verify candidate consistency is becoming even greater.

In this study, based on the previously reported calibration‐free NBED analysis framework [37], we developed a practical (*n*,*m*) assignment protocol that integrates uncertainty‐based local candidate generation directly from the measured diffraction spacings with self‐consistency checks across multiple computational pathways, explicitly accounting for manual measurement errors and CNT tilt relative to the incident electron beam. For validation of the protocol, we used a subset of the publicly available simulated diffraction dataset associated with Ref. [50] and deposited in Zenodo [51]. To mimic practical variations in experimentally acquired diffraction patterns, whole‐image noise and central‐beam intensity were varied, and a fixed‐shape beam‐stop shadow was added with randomized position and orientation. Using a total of 2394 test images covering CNT diameters of 0.48–2.3 nm, chiral angles of 0°–30°, and tilt angles of 0.5°–16.5°, we demonstrated that the assignment accuracy under realistic conditions involving manual analysis improved markedly from 71.6% without the proposed correction to 97.7% with the correction. The resulting protocol and accuracy dataset provide a broadly applicable analysis strategy to improve the accuracy and reproducibility of SWCNT chirality assignment, thereby strengthening the reliability basis of characterization and evaluation, supporting continued advances in chirality‐selective synthesis.

## 2 Experimental Section

### 2.1 Acquisition of Equatorial‐Line Spot Spacing and Layer‐Line Spacings

For (*n*,*m*) assignment, three key spacings are measured from the diffraction pattern of a SWCNT: the spot spacing *δ*′ on the equatorial line, and two layer‐line spacings, *d*′_2_ and *d*′_3_, defined as the distances from the equatorial line to the non‐equatorial layer lines L_2_ and L_3_, respectively (Figure 1). In the method reported by Jiang et al., any two layer‐line spacings can in principle be used for the calculation [37]. In the present study, L_2_ and L_3_ layer lines were selected for determining *d*′_2_ and *d*′_3_ because they are first‐order hexagonal layer lines located relatively close to the beam center and typically provide strong diffraction signals.

**FIGURE 1:**
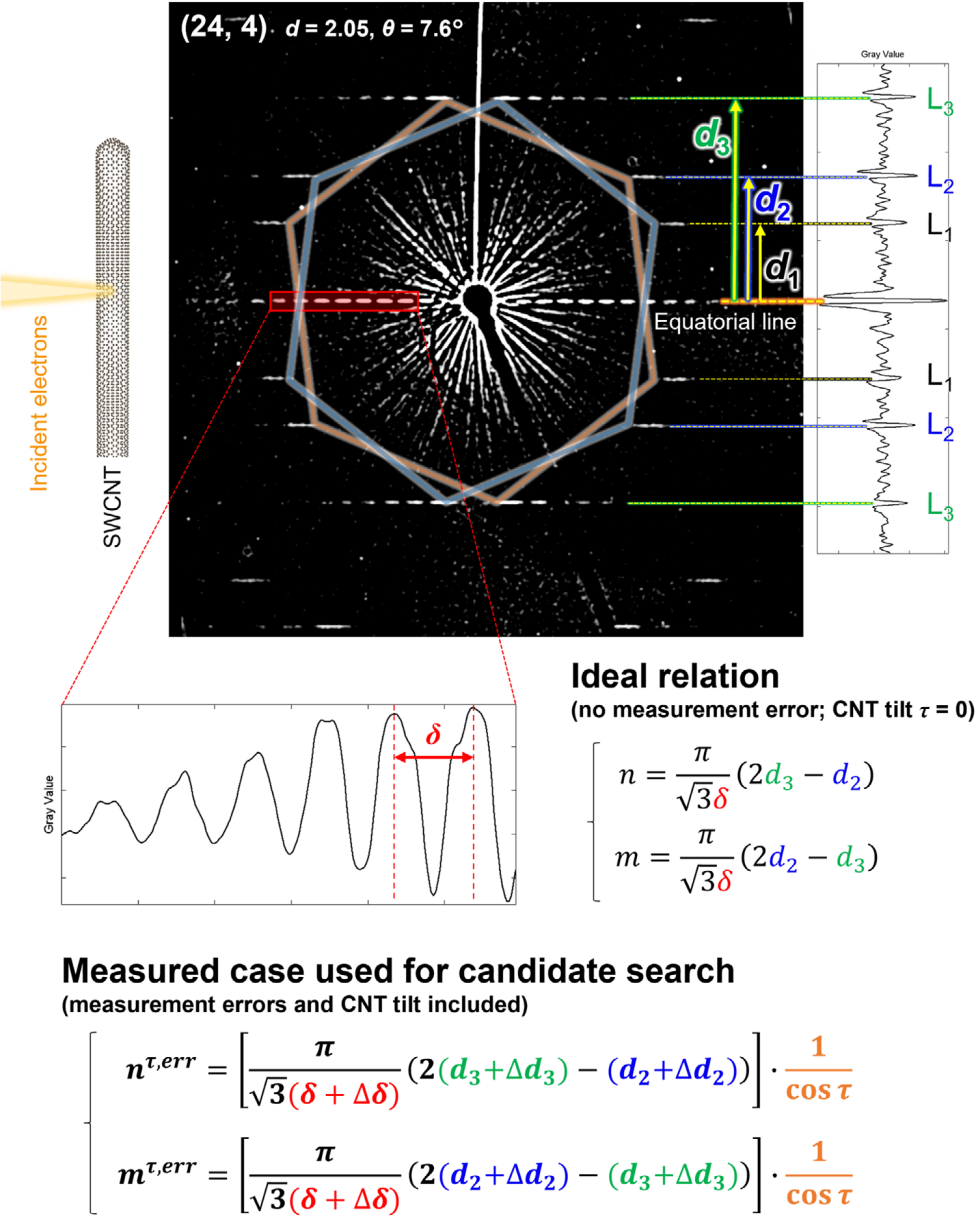
Experimentally acquired and preprocessed electron diffraction pattern of a SWCNT. The chiral indices (*n*,*m*) are assigned using three measured distance parameters: the pseudo‐periodic spot spacing *δ*′, defined as the peak‐to‐peak spacing in the intensity profile along the equatorial line, and the layer‐line spacings *d*′_2_ and *d*′_3_, defined as the distances from the equatorial line to the L_2_ and L_3_ layer lines, respectively. The lower equations illustrate the ideal case without manual measurement errors or CNT tilt, and the measured case in which the measured values *δ*′ = *δ* + Δ*δ*, *d*′_2_ = *d*_2_ + Δ*d*_2_, and *d*′_3_ = *d*_3_ + Δ*d*_3_ are affected by manual measurement errors and CNT tilt relative to the incident electron beam.

These distance parameters were measured manually from semiautomatically processed diffraction images using ImageJ. The measured equatorial‐line spacing *δ*′ was obtained from the intensity profile along the equatorial line. In the present study, 3–7 consecutive extrema were selected, and the median of the spacings between adjacent extrema was defined as *δ*′. This procedure reduced the influence of local errors in extrema‐position selection arising from the selection of a single pair of extrema. *d*′_2_ and *d*′_3_ were determined from the distances between the equatorial line and the corresponding L_2_ and L_3_ layer lines in fixed‐width line profiles drawn perpendicular to the equatorial line. The use of L_2_ and L_3_ allows a longer camera length to be employed while keeping the required layer lines within the detector field of view, thereby enabling the small oscillation spacing *δ*′ along the equatorial line to be sampled over a larger number of detector pixels. To facilitate visual inspection and manual measurement, several preprocessing steps were applied, including image denoising to smooth the intensity profiles, band‐pass filtering to reduce the influence of the direct electron beam (Figure S1), and image rotation prior to profiling to sharpen the layer‐line profiles. A workflow incorporating partial automation of these procedures was constructed using an ImageJ script, thereby standardizing image preprocessing, while the final peak selection and spacing measurements were performed manually with visual confirmation by the analyst.

### 2.2 (*n*,*m*) Assignment Protocol for CNT Diffraction Patterns Considering Manual Measurement Errors and CNT Tilt

When accurate values of *δ*, *d*_2_, and *d*_3_ are ideally obtained without error, the chiral indices (*n*,*m*) can be estimated from the following two fundamental equations:

(1) $$n=\frac{\pi }{\sqrt{3}\delta }\left(2d_{3}-d_{2}\right),m=\frac{\pi }{\sqrt{3}\delta }\left(2d_{2}-d_{3}\right)$$

However, experimentally acquired images may contain changes in the layer‐line spacings caused by the CNT tilt angle *τ*, defined here as the tilt of the nanotube axis relative to the incident electron beam [36]. When this CNT tilt effect is taken into account, the chirality‐related parameters become real‐valued quantities, expressed as *n^τ^* = *n*/cos*τ* and *m^τ^* = *m*/cos*τ*. In addition, the distance parameters measured manually inevitably contain manual measurement errors, Δ*δ*, Δ*d*_2_, and Δ*d*_3_, such that the measured values are given by *δ*′ = *δ* + Δ*δ*, *d*′_2_ = *d*_2_ + Δ*d*_2_, and *d*′_3_ = *d*_3_ + Δ*d*_3_, respectively. Accordingly, the chirality‐related parameters that include both tilt and manual measurement errors, *n^τ^* ^,err^ and *m^τ^* ^,err^, can be written as follows:

(2) $$\begin{array}{c} n^{\tau ,\mathrm{err}}=\left[\frac{\pi }{\sqrt{3}\delta^{\prime }}\left(2{d^{\prime }}_{3}-{d^{\prime }}_{2}\right)\right]\cdot \frac{1}{\cos\tau }\;\;\\ m^{\tau ,\mathrm{err}}=\left[\frac{\pi }{\sqrt{3}\delta^{\prime }}\left(2{d^{\prime }}_{2}-{d^{\prime }}_{3}\right)\right]\cdot \frac{1}{\cos\tau }\;\; \end{array}$$

In the method reported previously by Jiang et al., the real‐valued parameters *n^τ^* and *m^τ^* were converted into the final integer indices *n* and *m* using the TRUNC function, which removes the fractional part of a real number (Step 1 in Figure 2) [37]. However, because CNT tilt increases the real‐valued parameters *n^τ^* and *m^τ^* relative to the true integer indices *n* and *m*, simple truncation does not always recover the correct indices. When the tilt is sufficiently large, particularly for CNTs with large chiral indices, TRUNC(*n^τ^*) and TRUNC(*m^τ^*) can overestimate the true indices by one or more units. Therefore, the final integer indices cannot always be determined by a simple TRUNC operation alone, and it is necessary to consider whether the truncated values should be shifted downward by one or more units. In practical analysis of experimental diffraction images, no absolute criterion is available for determining this shift. When manual measurement errors are further included, the uncertainty becomes even greater, making precise narrowing of the candidates around *n^τ^* ^,err^ and *m^τ^* ^,err^ difficult.

**FIGURE 2:**
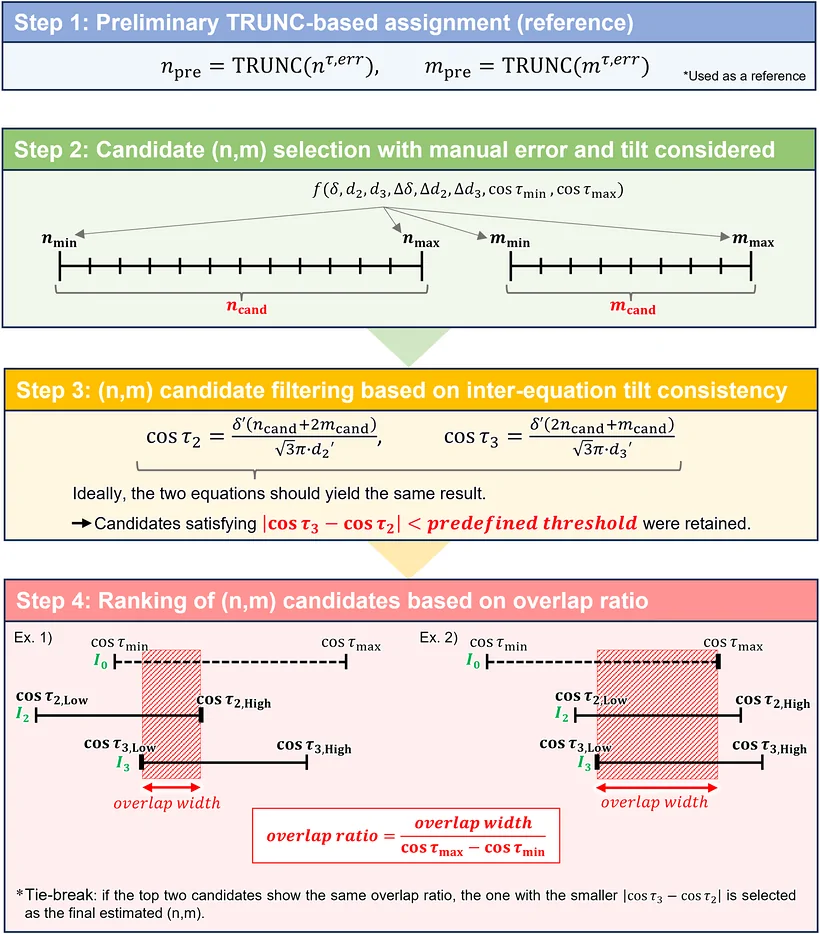
Workflow for (*n*,*m*) assignment from SWCNT diffraction patterns considering manual measurement errors and CNT tilt relative to the incident electron beam.

To address this issue, we propose a local candidate search protocol for (*n*,*m*) assignment that explicitly accounts for both CNT tilt and manual measurement errors as sources of uncertainty. First, continuous lower and upper bounds for *n* and *m* are directly calculated from the measured values *δ*′, *d*′_2_, and *d*′_3_, together with their assumed uncertainties and the allowable CNT tilt range. All integer pairs (*n*_cand_, *m*_cand_) within these calculated ranges are then enumerated under the condition 0 ≤ *m* ≤ *n* and defined as the candidate set (Step 2 in Figure 2). If the experimentally accessible diameter range of SWCNTs is known from other characterization methods, such as TEM observation, additional diameter‐based constraints can also be imposed on (*n*_cand_, *m*_cand_). Notably, (*n*_pre_, *m*_pre_), calculated in Step 1 of Figure 2, represents the reference assignment obtained using the conventional TRUNC‐based method and is not used in the candidate‐range calculation.

The tilt parameter can be obtained through multiple computational pathways, and these independently calculated values should, in principle, be identical because they are derived from the same CNT sample [37]. Based on this assumption, cross‐checking can be applied. In the present protocol, two forms of cos*τ*_i_, namely cos*τ*_2_ and cos*τ*_3_, were used:

(3) $$\begin{array}{c} \cos\tau_{2}=\frac{\delta^{\prime }\left(n_{\mathrm{cand}}+2m_{\mathrm{cand}}\right)}{\sqrt{3}\pi \cdot {d^{\prime }}_{2}}\;\;\\ \cos\tau_{3}=\frac{\delta^{\prime }\left(2n_{\mathrm{cand}}+m_{\mathrm{cand}}\right)}{\sqrt{3}\pi \cdot {d^{\prime }}_{3}}\;\; \end{array}$$

A two‐step narrowing of the candidate chiral indices was then performed based on Equation (3) as cross‐check‐based candidate filtering and ranking. In the initial screening step, only candidates satisfying |cos*τ*_3_ – cos*τ*_2_| below a predefined threshold were passed to the next step (Step 3 in Figure 2). For each remaining candidate (*n*_cand_, *m*_cand_), three allowable ranges of the tilt parameter cos*τ* were considered: the predefined calculated‐cos*τ* tolerance window, *I*_0_ = [cos*τ*_min_, cos*τ*_max_], and two candidate‐specific ranges, *I*_2_ = [cos*τ*_2,low_, cos*τ*_2,high_] and *I*_3_ = [cos*τ*_3,low_, cos*τ*_3,high_], estimated from Equation (3) using the predefined manual measurement error ranges. The overlap width was then defined as the width of the common intersection of these three ranges, *I*_0_ ∩ *I*_2_ ∩ *I*_3_. In other words, the overlap width represents the range of cos*τ* values for which a given candidate chirality is simultaneously consistent with the predefined calculated‐cos*τ* tolerance window and with both independently calculated tilt constraints derived from *d*_2_ and *d*_3_. The overlap ratio was obtained by normalizing this common overlap width by the width of the predefined tilt range:

(4) $$\mathrm{over}\mathrm{lap}\;\mathrm{ratio}=\frac{\mathrm{over}\mathrm{lap}\;\mathrm{width}}{\cos\tau_{\max}-\cos\tau_{\min}}$$

A larger overlap ratio therefore indicates that the candidate remains physically consistent over a wider range of possible CNT tilt values, even when manual measurement errors are considered. Using this overlap ratio, all candidates that passed Step 3 were ranked (Step 4 in Figure 2). When two or more candidates showed the same overlap ratio, a tie‐break rule was applied so that the candidate with the smaller |cos*τ*_3_ – cos*τ*_2_| was prioritized.

Here, the local candidate search refers to the enumeration of integer (*n*,*m*) pairs that satisfy the predefined uncertainty windows for the measured spacings and the calculated CNT‐tilt parameter. In this study, the search space was defined by Δ*δ* = ±0.0150*δ*′, Δ*d*_2_ = ±0.0075*d*′_2_, Δ*d*_3_ = ±0.0075*d*′_3_, the calculated cos*τ* tolerance window of 0.94–1.02, and the diameter constraint of *d*_CNT_ < 4.0 nm, as summarized in Table S1. Thus, the search was restricted to candidate chiralities consistent with the predefined measurement‐error and CNT‐tilt uncertainty windows derived from the measured *δ*′, *d*′_2_, and *d*′_3_, rather than being performed over the full (*n*,*m*) space.

These analytical procedures were implemented and validated using an Excel Visual Basic for Applications (VBA) macro integrated with the ImageJ‐based image‐analysis workflow described in Section 2.1. The Excel‐based analysis tool and a detailed user guide are provided in the Supporting Information. In the conventional approach, the analysis, including part of the cross‐check procedure, was performed manually and required approximately 15 min per diffraction pattern. In contrast, the present integrated system reduced the analysis time to approximately 0.5–1 min per diffraction pattern.

The main predefined parameters used in this study are summarized in Table S1. These parameters were optimized through preliminary validation. The results of parameter‐sensitivity analyses, in which the relative‐error parameter *ε*, cos*τ*_min_, and the threshold of |cos*τ*_3_ − cos*τ*_2_| were varied independently, are shown in Figure S2. Here, *ε* defines the allowable relative‐error ranges as Δ*δ* = ±*εδ*′, Δ*d*_2_ = ±0.5*ε* *d*′_2_, and Δ*d*_3_ = ±0.5*ε* *d*′_3_. The assignment accuracy showed a non‐monotonic, peaked dependence on *ε*. At *ε* = 0.090, frequent tie‐breaks occurred and the assignment accuracy decreased to approximately 70% (Figure S2a). Increasing *ε*, that is, excessively widening the allowable error ranges for the layer‐line spacings *d*_2_ and *d*_3_ and the equatorial‐line spot spacing *δ*, not only increases the number of potential chiralities included in the candidate set but also broadens the candidate‐specific ranges *I*_2_ and *I*_3_ derived from *d*_2_ and *d*_3_. Consequently, their common overlap with *I*_0_ can readily extend across the full width of *I*_0_, causing the overlap ratio normalized by *I*_0_ to saturate at its upper limit of 1. Indeed, when a (21,9) CNT tilted by 16.5° was analyzed with *ε* = 0.090, three candidate chiralities exhibited an overlap ratio of 1, and after application of the tie‐break rule, (24,10), an incorrect chirality with substantially different *n* and *m* values, was ultimately assigned. Conversely, when *ε* is too small in the presence of manual measurement errors, even the correct chirality may fall outside the allowable range, resulting in final assignment to an incorrect neighboring chirality and a corresponding decrease in accuracy.

A similar non‐monotonic, peaked dependence of assignment accuracy was observed for cos*τ*_min_, which determines the upper limit of the allowable CNT tilt angle (Figure S2b). If cos*τ*_min_ is set too high, corresponding to an excessively low allowable tilt angle, CNTs with actual tilt angles beyond this range may not be correctly assigned. Conversely, if cos*τ*_min_ is set too low, corresponding to an excessively large allowable tilt angle, the candidate set cannot be sufficiently narrowed, resulting in reduced assignment accuracy. For the threshold of |cos*τ*_3_ − cos*τ*_2_|, a large number of no‐valid‐candidate cases occurred at low threshold values, suggesting that this parameter should be set within an appropriate range that allows effective candidate narrowing without excessively excluding valid candidates (Figure S2c).

These predefined parameters may need to be adjusted depending on factors that affect the measurement uncertainty of *δ*, *d*_2_, and *d*_3_, such as camera length, detector pixel size or binning, signal‐to‐noise ratio, exposure time, direct‐beam saturation, and background intensity from the support film or contamination. In addition, the allowable window for the calculated cos*τ* values and the diameter constraint should be adjusted according to the expected CNT tilt range and the independently estimated diameter range of the analyzed SWCNT sample. In particular, when the sample contains CNTs with large tilt angles, candidate narrowing becomes less effective. Moreover, if the actual tilt angle exceeds the assumed range, there is a risk that the user may not recognize the resulting misassignment because no dedicated criterion is currently implemented to identify such out‐of‐range tilt conditions. Therefore, careful sample preparation is desirable, including minimizing bending of the TEM grid and, for example, depositing a few drops of toluene or acetone onto the CNT‐loaded TEM grid and allowing it to dry so that the CNTs are brought into closer contact with the grid surface [14, 50].

Regarding user‐to‐user variability, the selection of diffraction spots and line positions is based on peak positions identified from image‐intensity profiles, as illustrated in Figure 1. In addition, *δ*′ is determined from the median of the spacings between multiple consecutive extrema, while *d*′_2_ and *d*′_3_ are determined by averaging the corresponding layer‐line distances measured on both sides of the equatorial line. Therefore, the resulting variability is expected to be relatively small, and minor measurement errors can be accommodated by appropriately setting the allowable error ranges for the layer‐line spacings *d*_2_ and *d*_3_ and the equatorial‐line spot spacing *δ*. In contrast, as discussed above, the sample tilt condition may introduce substantially larger, user‐unrecognized variability or error. Particular attention should therefore be paid to minimizing sample tilt during specimen preparation.

### 2.3 Generation of Test Diffraction Images Mimicking Experimentally Acquired Patterns

For comparison with the simulated diffraction patterns described below, a representative experimentally acquired NBED pattern is shown in Figure 3a. For validation, we used a subset of the publicly available simulated diffraction dataset associated with Ref. [50] and deposited in Zenodo [51]. The subset comprised 266 SWCNT chiralities with diameters of 0.48–2.3 nm and chiral angles of 0°–30°, at tilt angles ranging from 0.5° to 16.5° in 2° increments, resulting in 2394 experiment‐mimicking diffraction images. The detailed simulation and image‐generation procedures are described in Ref. [50]. The images used in the present validation incorporated experimentally relevant perturbations, including whole‐image noise, variations in central direct‐beam intensity, diffraction‐pattern rotation, and randomized beam‐stop position and orientation (Figure 3). All 2394 images were used for evaluation under each analysis condition.

**FIGURE 3:**
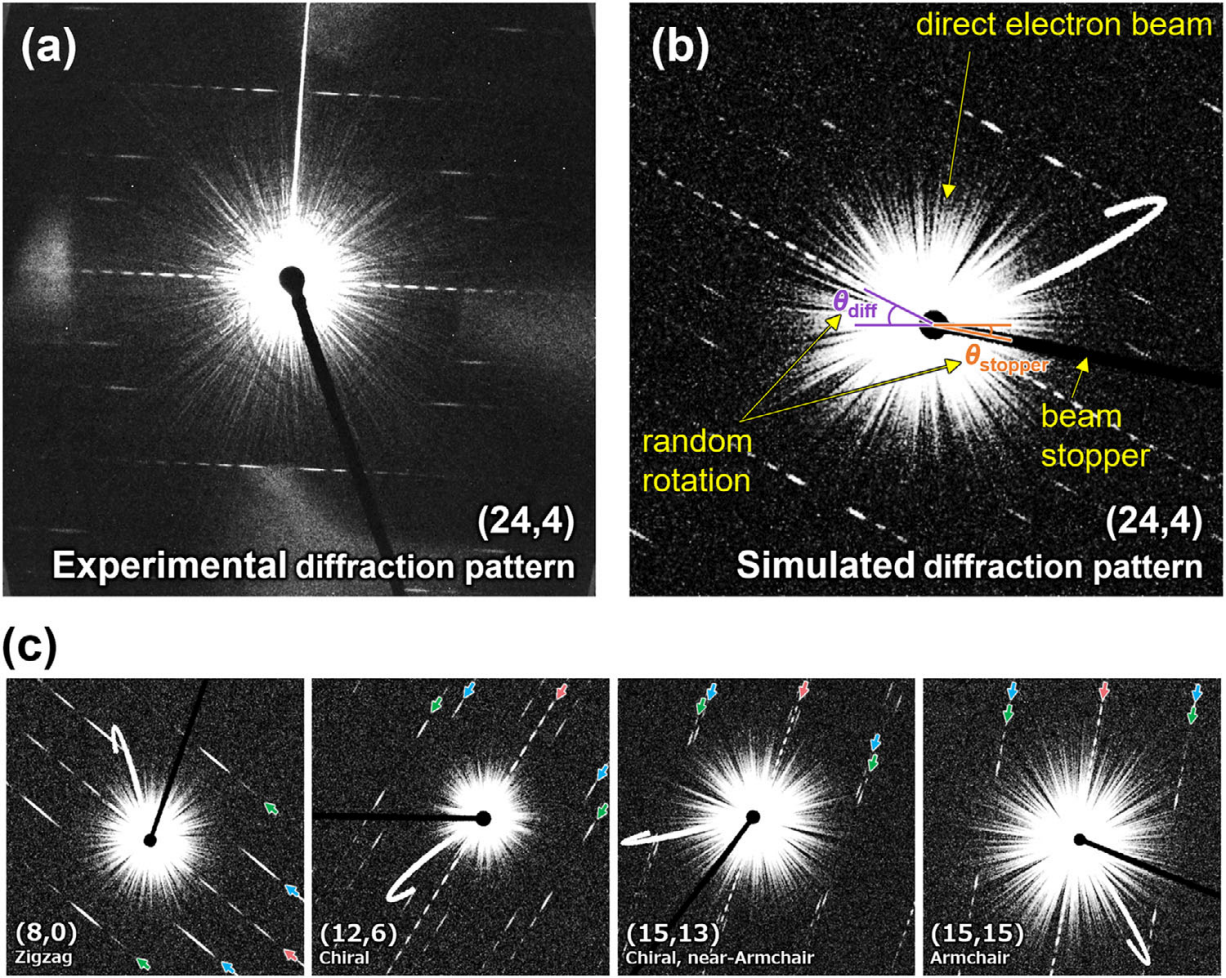
(a) Experimental and (b) simulated diffraction patterns of a SWCNT. To mimic experimentally acquired diffraction images, whole‐image noise, central direct‐beam intensity, diffraction‐pattern rotation, and a fixed‐shape beam‐stop shadow with randomized position and orientation were added to the simulated diffraction images to generate the test dataset. (c) Representative simulated test diffraction patterns of SWCNTs with different chiralities. Colored arrows in panel (c) indicate the equatorial line (red), L_2_ (blue), and L_3_ (green).

### 2.4 Experimental NBED Dataset for Comparison With AI Predictions

For a complementary experimental consistency assessment, we reused the experimental NBED dataset previously reported in Ref. [50]. Detailed experimental procedures are described in Ref. [50], with additional details of the SWCNT synthesis conditions provided in the corresponding original studies [8, 14, 18, 27, 52, 53]. In the present study, the same experimental NBED images were reanalyzed using the manual assignment protocol described above, both without and with cross‐check correction. AI predictions obtained using the ViT‐Large model reported in Ref. [50] were used as provisional reference labels. To increase the reliability of this comparison, only patterns with an AI prediction confidence score of 0.85 or higher were included, resulting in an evaluation dataset of 568 NBED patterns.

## 3 Results

### 3.1 Evaluation of Chirality‐Assignment Accuracy Using Simulated Diffraction Images

To compare the accuracy of the present analysis protocol, the results obtained without cross‐check correction (w/o corr.) were compared with those obtained with cross‐check correction (w/ corr.), as summarized in Figure 4. In the analysis without cross‐check correction, the values calculated from the fundamental equations in Equation (1) were directly converted into integers using the TRUNC function (Step 1 in Figure 2). In contrast, the analysis with cross‐check correction used the local candidate search protocol shown in Figure 2, including candidate generation, self‐consistency screening, and overlap‐ratio‐based ranking (Steps 1–4). The overall assignment accuracy improved markedly from 71.6% without correction to 97.7% with correction, corresponding to an enhancement of more than 26 percentage points. Regardless of whether cross‐check correction was applied, the deviations in *n* and *m* remained within ±1. This clearly indicates that the proposed method, which enumerates candidate chiralities directly from the uncertainty ranges defined by the manually measured *δ*′, *d*′_2_, and *d*′_3_ values and subsequently applies cross‐check‐based filtering and ranking, is effective for accurate identification of the final candidate.

**FIGURE 4:**
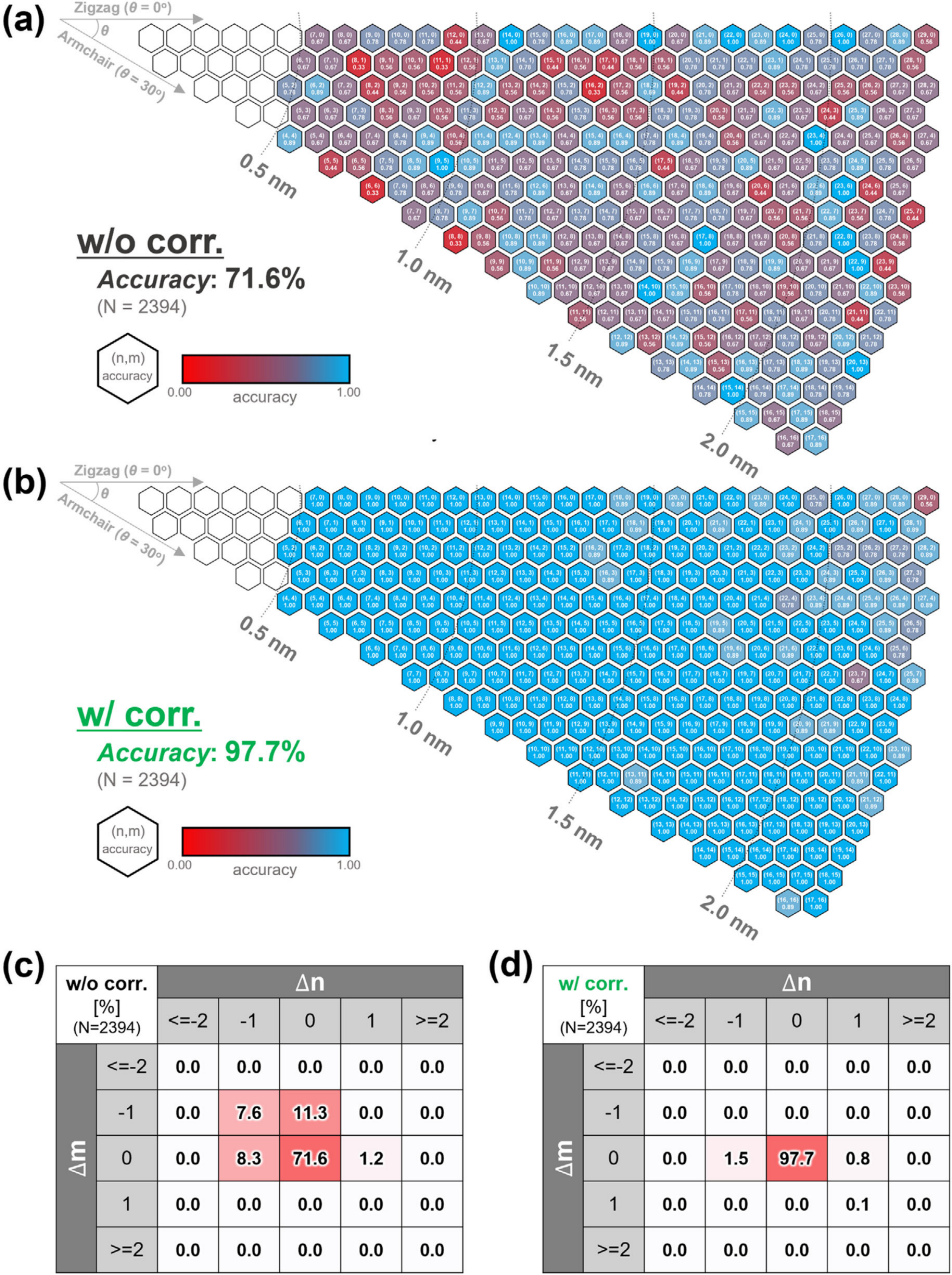
Color‐coded (*n*,*m*) assignment accuracy plotted on the SWCNT chirality map for analyses performed (a) without cross‐check correction and (b) with cross‐check correction. Each hexagon represents a specific (*n*,*m*) species, and the color indicates the fraction of correctly assigned diffraction patterns for that species. Lighter blue denotes higher accuracy, whereas red denotes lower accuracy. (c,d) Distributions of assignment errors in *n* and *m*, expressed as Δ*n* and Δ*m*, for analyses performed (c) without cross‐check correction and (d) with cross‐check correction.

In practice, chirality assignment is often used to evaluate the mean values and distributions of CNT diameter, chiral angle, as well as the metallic CNT fraction. The errors in these dataset‐level structural parameters are summarized in Table 1. For the case without cross‐check correction, the error rates in the mean CNT diameter, mean chiral angle, and metallic CNT fraction were −1.33%, −2.12%, and −2.75%, respectively. In contrast, for the case with cross‐check correction, the corresponding error rates were −0.03%, +0.02%, and ±0.00%, respectively, showing excellent agreement with the dataset‐level ground‐truth values.

**TABLE 1:** Errors in dataset‐level SWCNT structural parameters derived from (*n*,*m*) assignment.

Dataset‐level structural parameter	Ground‐truth value	w/o corr.		w/ corr.	
		Analysis results	Error	Analysis results	Error
Mean CNT diameter [nm]	1.57	1.55	−0.02 (−1.33%)	1.57	−0.00 (−0.03%)
Mean chiral angle [deg.]	14.66	14.35	−0.31 (−2.12%)	14.66	+0.00 (+0.02%)
Metallic CNT fraction [%]	34.96	34.00	−0.96 (−2.75%)	34.96	±0.00 (±0.00%)

To further analyze the effects of the cross‐check correction, additional analyses were performed using only tilt‐parameter consistency, defined by minimization of |cos*τ*_3_ – cos*τ*_2_| (Step 3 in Figure 2), or only overlap‐ratio maximization (Step 4 in Figure 2) for candidate selection. When the candidates were ranked solely on the basis of the consistency between cos*τ*_2_ and cos*τ*_3_, the assignment accuracy was only 47.1%, which was even lower than that obtained without cross‐check correction (Figure S3). In addition, a certain fraction of the results showed deviations in Δ*n* and Δ*m* of 2 or more, indicating that simple evaluation based only on tilt‐parameter consistency was not sufficient for effective narrowing of candidates during the local candidate search. In contrast, when chirality candidates were ranked using only the overlap ratio, the assignment accuracy reached 96.7%, and all Δ*n* and Δ*m* values remained within ±1 (Figure S4). Although this value was approximately 1 percentage point lower than the 97.7% obtained when overlap‐ratio‐based ranking and tilt‐parameter consistency were combined, it still indicates sufficiently high accuracy. These results indicate that the overlap ratio newly introduced in this study plays a central role in final candidate selection in the manual analysis of NBED patterns.

To further investigate the origin of misclassification, the dependence of assignment error on CNT diameter and chiral angle was examined. For each individual CNT, the assignment error was defined as |Δ*n*| + |Δ*m*|, where Δ*n* and Δ*m* are the differences between the assigned and ground‐truth chiral indices. The values plotted in Figure 5 represent the mean assignment error averaged within each CNT‐diameter or chiral‐angle bin. For the case without cross‐check correction, the mean assignment error showed a gradual decreasing trend with increasing CNT diameter, although the difference between the maximum and minimum values remained less than twofold (Figure 5a). Similarly, little dependence of the mean assignment error on chiral angle was observed (Figure 5b). In contrast, for the case with cross‐check correction, although the absolute values of the mean assignment error were approximately one order of magnitude smaller than those without cross‐check correction, the bin‐averaged error was zero for small‐diameter CNTs, began to increase from around 1.4 nm, and rose markedly toward the largest diameter range in the test dataset, around 2.2 nm (Figure 5c). A similar trend was observed with respect to chiral angle, where the mean assignment error increased as the chiral angle decreased, that is, as the structure approached the zigzag type (Figure 5d).

**FIGURE 5:**
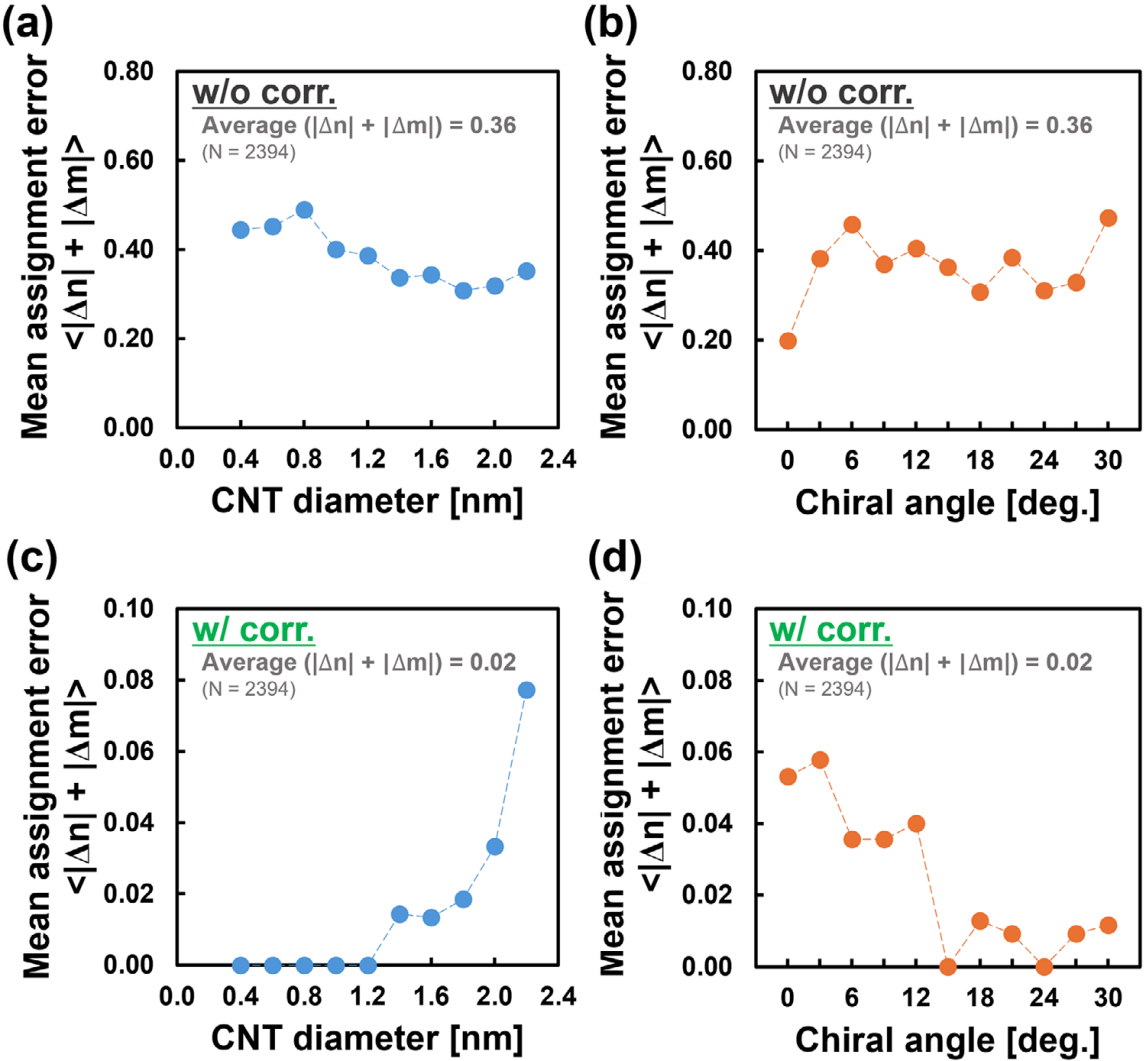
Dependence of the mean assignment error, defined as the bin‐averaged value of |Δ*n*| + |Δ*m*|, on CNT diameter ((a) without and (c) with cross‐check correction) and on chiral angle ((b) without and (d) with cross‐check correction).

### 3.2 Comparison of AI Predictions and Manual Chirality Assignments for Experimental NBED Images

In the preceding section, evaluation using 2394 simulated diffraction patterns showed that the proposed protocol correctly assigned 97.7% of the test patterns. Although perturbations such as image noise, variations in central‐beam intensity, random image rotation, and beam‐stop shadows were introduced into the simulated images, simulated patterns cannot fully reproduce the complexity of real experimental conditions. In practical TEM measurements, additional factors, including axial bending of CNTs, amorphous scattering from the substrate, diffraction‐spot broadening, beam drift, and amorphous carbon contamination on CNT walls, may further degrade the measurement accuracy of layer‐line and spot spacings.

Despite the importance of assessing the method using experimentally acquired images, such an assessment is complicated by the lack of a broad experimental NBED dataset paired with independently verified ground‐truth chirality labels. Several established methods for chirality assignment from NBED patterns have been reported [36, 37, 44, 45, 50]; however, their experimental validation has generally been demonstrated using limited sets of CNTs rather than a common ground‐truth dataset covering a broad range of chiralities and tilt conditions [36, 37, 44, 45]. In addition, different methods have different applicability requirements, such as assumptions regarding CNT tilt or the need for instrumental calibration. Such datasets are therefore insufficient as a comprehensive experimental benchmark for the present study, which aims to establish a reproducible assignment framework applicable to a broad range of experimentally synthesized CNT chiralities.

To provide a complementary evaluation using experimental NBED images, we reused the experimental dataset employed to validate a recently developed AI‐based chirality classifier [50]. This AI classifier was trained entirely on simulated diffraction images and achieved an accuracy of 99.96% for simulated test images. For the full experimental dataset of 909 patterns, the model showed 79.8% exact agreement and 99.3% near‐neighbor agreement with manual chirality assignments, where near‐neighbor agreement allowed deviations of up to ±1 in both *n* and *m*. In the present analysis, AI predictions were used as provisional reference labels. To increase the reliability of these labels, the evaluation dataset was restricted to predictions with an AI confidence score of 0.85 or higher. The resulting dataset consisted of 568 experimental NBED images covering a broad range of CNT chiralities, with diameters of 0.96–2.29 nm and chiral angles of 0°–30°.

Figure 6 compares the manual assignments with the AI predictions for this dataset. Without cross‐check correction, the exact agreement and near‐neighbor agreement with the AI predictions were 58.1% and 99.1%, respectively. With cross‐check correction, these values increased to 86.6% and 99.5%, respectively. The relationship between these agreement values and those previously reported in Ref. [50] is provided in Table S2. Because the AI predictions served as provisional reference labels rather than independently verified ground truth, the values in Figure 6 are reported as agreement metrics rather than absolute assignment accuracies. The dataset‐level structural parameters derived from the AI predictions and manual assignments are summarized in Table 2. For both the mean CNT diameter and mean chiral angle, the values obtained from the manual assignments were within 1% of those derived from the AI predictions. The metallic CNT fraction differed by approximately 2.1 percentage points between the AI predictions and manual assignments.

**FIGURE 6:**
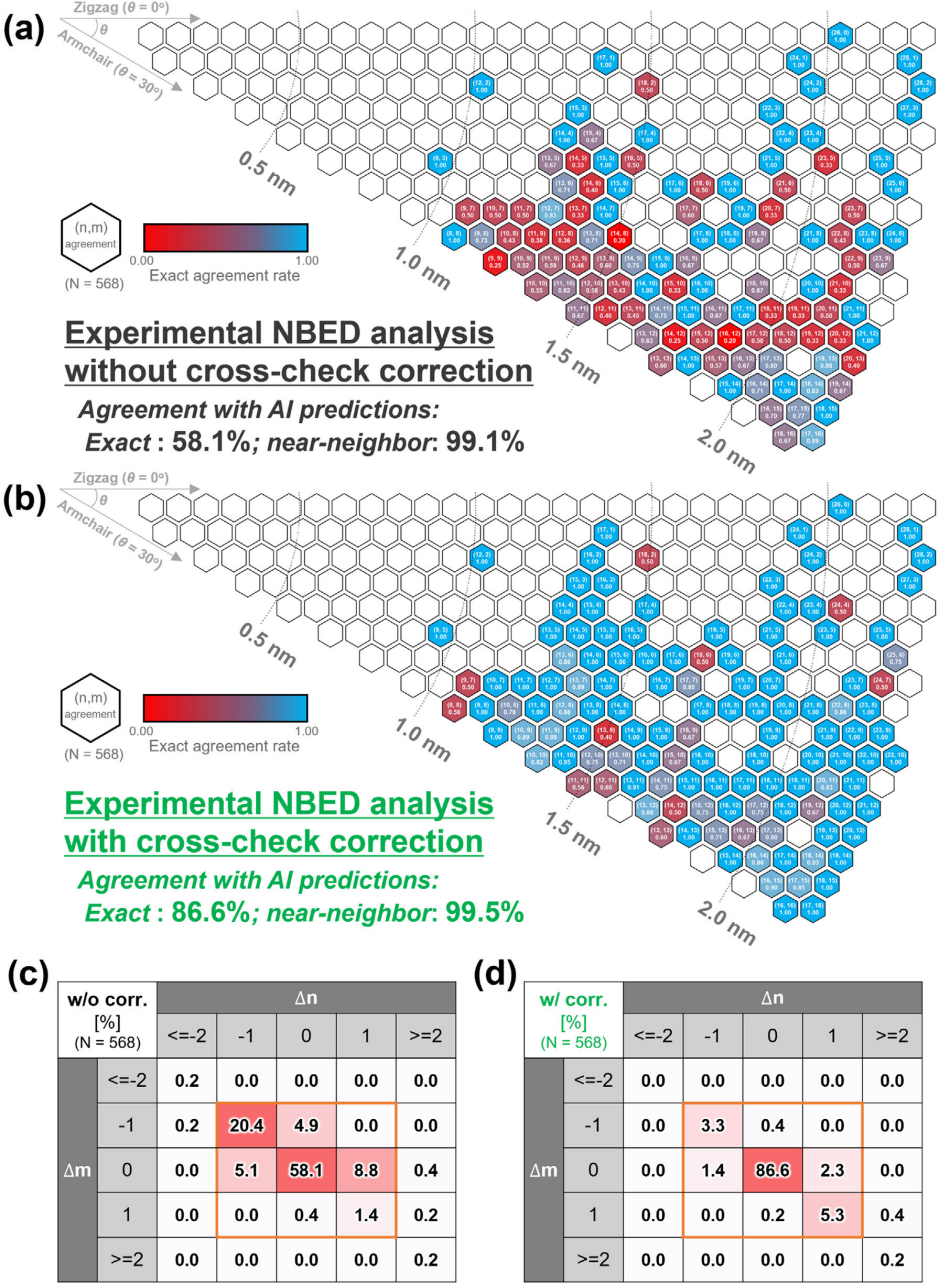
Comparison between AI predictions and manual chirality assignments for 568 experimental NBED patterns. (a,b) Chirality‐resolved exact agreement for manual assignments obtained (a) without and (b) with cross‐check correction. Each hexagon represents a specific (*n*,*m*) species, and the color indicates the fraction of manual assignments that exactly matched the AI predictions for that species. The numbers in each hexagon indicate the exact agreement rate for that species. (c,d) Distributions of differences in *n* and *m*, expressed as Δ*n* and Δ*m*, between the manual assignments and AI predictions for analyses performed (c) without and (d) with cross‐check correction. The orange boxes indicate the near‐neighbor region (|Δ*n*| ≤ 1 and |Δ*m*| ≤ 1) used to calculate the near‐neighbor agreement.

**TABLE 2:** Comparison of dataset‐level SWCNT structural parameters derived from AI predictions and manual chirality assignments for 568 experimental NBED patterns.

Dataset‐level structural parameter	AI predictions	Manual assignments	
		w/o corr.	w/ corr.
Mean CNT diameter [nm]	1.66	1.64	1.67
Mean chiral angle [deg.]	22.93	22.73	22.93
Metallic CNT fraction [%]	26.41	28.52	28.52

## 4 Discussion

### 4.1 Why the Local Candidate Search Protocol Functioned Effectively

The local candidate search protocol proposed in this study should be understood not as a method for reconstructing chirality assignment from scratch, but as a framework for selecting the most plausible candidate from multiple integer (*n*,*m*) pairs generated within uncertainty‐defined ranges derived directly from the manually measured *δ*′, *d*′_2_, and *d*′_3_ values. In fact, regardless of whether cross‐check correction was applied, the final deviations in *n* and *m* remained within ±1. This result indicates that, even in the presence of manual measurement errors, the conventional TRUNC‐based preliminary assignments generally remained in the neighborhood of the true chirality.

Therefore, the essential challenge is to robustly discriminate among competing local candidates rather than to search for the correct solution over a broad (*n*,*m*) space. From this perspective, the protocol shown in Figure 2 accommodates small deviations caused by manual measurement errors and CNT tilt uncertainty by enumerating candidate chiralities permitted by the predefined uncertainty windows and ranking them using cross‐check‐based consistency criteria. Although a certain level of accuracy was obtained even without cross‐check correction, the improvement to 97.7% with correction suggests that the primary error source is misidentification among neighboring candidate chiralities within the local uncertainty window, rather than a large deviation of the preliminary estimate from the true chirality.

### 4.2 Different Roles of the Overlap Ratio and Tilt‐Parameter Consistency

Comparison of the individual components of the cross‐check correction revealed that the use of tilt‐parameter consistency alone, defined here as the agreement between cos*τ*_2_ and cos*τ*_3_ independently calculated from *d*_2_ and *d*_3_, resulted in an accuracy of only 47.1%, which was even lower than that obtained without correction. In contrast, candidate ranking based solely on the overlap ratio achieved a high accuracy of 96.7%, with all Δ*n* and Δ*m* values remaining within ±1. This clear contrast indicates that the two metrics play different roles in candidate selection.

The tilt‐parameter consistency criterion based on |cos*τ*_3_ − cos*τ*_2_| evaluates how closely the two point estimates of the tilt parameter, independently calculated from *d*_2_ and *d*_3_, agree with each other. It is therefore useful as an initial screening criterion for excluding candidates for which the two estimates differ substantially. However, this metric evaluates only the difference between the two point estimates and does not consider whether they lie within the physically allowable tilt range or whether their consistency is maintained when measurement errors are taken into account.

This difference is illustrated in Figure 7 using the actual calculation results for a simulated (24,4) CNT tilted by 6.5°, which was included in the validation dataset. As shown in Figure 7a, as the candidate indices vary, local combinations in which cos*τ*_2_ and cos*τ*_3_ approach each other occur repeatedly. Consequently, multiple neighboring candidates can exhibit similarly small values of |cos*τ*_3_ − cos*τ*_2_|. Because these local minima are narrow and steep, small shifts in the manually measured equatorial‐line spot spacing or layer‐line positions can readily alter their relative depths and hence the candidate ranking. Indeed, in the example shown, the incorrect candidate (25,4) exhibits a slightly smaller |cos*τ*_3_ − cos*τ*_2_| than the correct candidate (24,4). Thus, minimizing this difference alone ranks the incorrect candidate above the correct one. This example demonstrates that agreement between the two tilt estimates alone does not provide sufficient information for unique final candidate selection.

**FIGURE 7:**
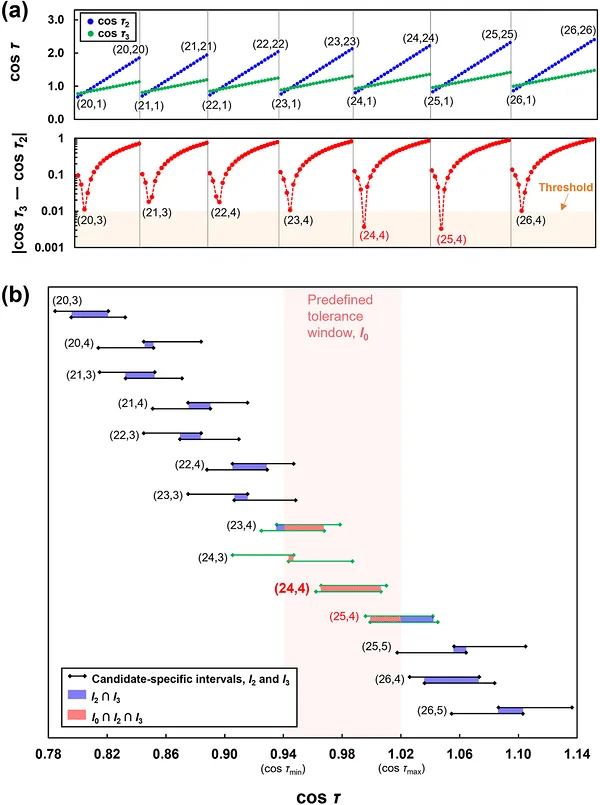
Comparison of tilt‐parameter consistency and overlap‐ratio evaluation using a simulated (24,4) CNT tilted by 6.5°. (a) Candidate‐dependent values of cos*τ*_2_ and cos*τ*_3_ (top) and |cos*τ*_3_ − cos*τ*_2_| (bottom), plotted for all integer (*n*,*m*) combinations satisfying *n* = 20–26 and 0 ≤ *m* ≤ *n*, including candidates that would normally be excluded during the filtering process. The orange‐shaded region in the lower panel indicates values satisfying the predefined |cos*τ*_3_ − cos*τ*_2_| threshold. (b) Candidate‐specific intervals *I*_2_ and *I*_3_ for the subset of candidates in (a) for which *I*_2_ and *I*_3_ overlap (nonempty *I*_2_ ∩ *I*_3_). Blue segments indicate *I*_2_ ∩ *I*_3_, red segments indicate *I*_0_ ∩ *I*_2_ ∩ *I*_3_, and the shaded region represents the predefined tolerance window *I*_0_.

In contrast, the overlap ratio evaluates the candidate‐specific tilt‐parameter intervals *I*_2_ and *I*_3_ obtained by considering measurement errors, rather than only their point estimates. In Figure 7b, the blue segments indicate *I*_2_ ∩ *I*_3_, while the red segments indicate the common intersection with the predefined tolerance window, *I*_0_ ∩ *I*_2_ ∩ *I*_3_. A candidate with a larger overlap ratio can therefore be interpreted as remaining consistent with the constraints derived from both *d*_2_ and *d*_3_ over a wider range of allowable CNT tilt and measurement uncertainty. In the example shown, the correct candidate (24,4) has a wider three‐way common intersection than the incorrect candidate (25,4). Accordingly, whereas simple minimization of |cos*τ*_3_ − cos*τ*_2_| selects (25,4), overlap‐ratio ranking correctly prioritizes (24,4). In other words, tilt‐parameter consistency evaluates the agreement between two computational pathways at the level of point estimates, whereas the overlap ratio evaluates interval‐level consistency that incorporates both measurement uncertainty and the allowable CNT tilt range.

This distinction is consistent with the assignment accuracy of only 47.1% obtained using tilt‐parameter consistency alone, compared with 96.7% using the overlap ratio alone. Furthermore, combining the overlap ratio with tilt‐parameter consistency further increased the accuracy by approximately 1 percentage point to 97.7%. These results indicate that the overlap ratio serves as the primary criterion for final candidate ranking, whereas tilt‐parameter consistency is better suited for screening clearly inconsistent candidates and for complementary discrimination among candidates with identical rankings.

### 4.3 Discrepancies Between AI and Manual Assignments for Experimental NBED Images

As shown in Figure 6, the experimental consistency assessment yielded an exact agreement of 86.6% between the AI predictions and manual assignments. As described in Section 2.4, the AI predictions were used as provisional reference labels rather than independently verified ground truth. Both approaches achieved high assignment accuracy on simulated datasets with known chirality labels, with 99.96% reported for the ViT‐Large model [50] and 97.7% for the present manual protocol. Taken together, these results suggest that the lower exact agreement observed for experimental images may involve additional experimental factors or method‐specific limitations not fully represented in the simulated datasets. To investigate the origin of these discrepancies, we examined in detail the experimental patterns showing relatively large differences between the two assignments. The NBED image showing the largest assignment discrepancy within the present experimental comparison dataset is shown in Figure S5a. The maximum assignment difference, defined as |Δ*n*| + |Δ*m*|, was 5; this pattern was assigned as (16,8) by the AI model and as (19,10) by the manual analysis with cross‐check correction (Figure S5b).

The diffraction spots and layer lines in this image appear relatively clear, suggesting that poor imaging quality is unlikely to be the primary cause of the discrepancy. One distinctive feature is that the pair of diffraction spots associated with L_3_ approaches each other near the meridian passing through the diffraction center, as indicated by the yellow arrows in Figure S5a. Simulated diffraction patterns of the manually assigned (19,10) chirality at different tilt angles, without added noise, beam‐stop shadows, or other perturbations, are shown in Figure S5c–g. As the tilt angle increases, the corresponding pair of L_3_ spots progressively approaches each other and becomes nearly coincident at a tilt angle of 20°. This trend is also clearly observed in Figure S5h, which plots the distance between these spots as a function of CNT tilt angle. These observations suggest that the NBED pattern in Figure S5a, for which the AI and manual assignments showed a relatively large discrepancy, may have originated from a CNT with a comparatively large tilt angle, possibly around 20°.

The AI model used in the present comparison was trained using simulated diffraction patterns with tilt angles ranging from 0° to 20° [50] and therefore contained no training examples beyond this range. Likewise, in the manual analysis, cos*τ*_min_ was set to approximately cos20° = 0.94, corresponding to a maximum allowable CNT tilt angle of approximately 20°. The assignment reliability may therefore decrease for NBED patterns originating from CNTs tilted beyond this range. It is not possible from the present comparison to determine whether the large discrepancy in this particular case originated primarily from the AI prediction or from the manual assignment. Nevertheless, the inclusion of CNTs with large tilt angles beyond the ranges explicitly covered by either analysis may substantially reduce assignment reliability. This may be one factor contributing to the lower exact agreement, relative to near‐neighbor agreement, observed between the AI‐based and manual analyses of experimental NBED images.

### 4.4 Remaining Sources of Misclassification in the Large‐Diameter, Low‐Chiral‐Angle Region and Limitations of the Present Method

Although the overall accuracy was markedly improved by the correction, the classification error still exhibited clear dependence on CNT diameter and chiral angle. Specifically, the classification error was nearly zero for small‐diameter CNTs, but began to increase above approximately 1.4 nm and rose markedly in the largest‐diameter region of the test dataset, around 2.2 nm. A similar trend was observed for chiral angle: the error remained low on the armchair side but increased as the structure approached the zigzag side.

The main origin of this trend is likely that, for CNTs with large diameters and low chiral angles, the differences in spot spacings and layer‐line spacings between neighboring chiralities become smaller, causing the diffraction features of adjacent candidates to become increasingly similar [35, 45, 46]. Furthermore, in the presence of CNT tilt relative to the incident electron beam, the separability of these features is further reduced, and the diffraction features of neighboring candidates begin to partially overlap [35, 36, 37, 46]. As a result, even when manual measurement errors are small, the intrinsic margin for discrimination between candidates becomes narrower, making final misclassification more likely. In other words, although the present method is highly effective for correcting local errors originating from manual measurement, it does not completely eliminate the intrinsically difficult regions imposed by diffraction geometry itself. Therefore, assignments obtained in the large‐diameter, low‐chiral‐angle region should be interpreted more cautiously, considering that the intrinsic differences between neighboring candidates can become very small. For example, for CNTs assigned to this region, a more stringent analysis procedure could be adopted in which the extrema on the equatorial line and the layer‐line positions are independently reselected several times, followed by repeated analysis to confirm that the assignment remains stable against manual feature selection.

In contrast, no such pronounced trends were observed in the analysis without cross‐check correction. This difference between the corrected and uncorrected analyses suggests that, before correction, the overall error level was sufficiently large to obscure the underlying structural dependence of misclassification. After cross‐check correction reduced this background error, the intrinsic tendency for misclassification to increase in the large‐diameter, low‐chiral‐angle region became more clearly visible.

These observations clarify several directions for future improvement. In the large‐diameter, low‐chiral‐angle region, further refinement of the definition of the overlap ratio may enhance subtle differences between competing candidates. The protocol could also be extended to incorporate diffraction‐intensity information, particularly from the equatorial line, which exhibits intensity oscillations governed by the zeroth‐order Bessel function and reflects diameter information [35, 36]. In the present protocol, the median spacing between adjacent extrema is calculated from 3–7 consecutive extrema in the equatorial‐line profile and used as the representative value of *δ*′. Although this approach reduces the influence of local measurement errors compared with relying on a single pair of extrema, it compresses the entire profile into a single spacing parameter, leaving the remaining information contained in the oscillation shape unused for candidate selection. For an idealized straight SWCNT, the equatorial‐line intensity modulation is governed primarily by the zeroth‐order Bessel function, and its spatial periodicity reflects the CNT diameter while remaining independent of rigid CNT tilt [36, 37]. Therefore, a possible future extension would be to fit the entire background‐corrected equatorial‐line profile with a $J_{0}^{2}$‐based model and estimate a common oscillation scale from multiple visible oscillations, thereby enabling a more robust determination of *δ*′. If the experimental uncertainty in *δ*′ can be reduced, a narrower relative‐uncertainty window *ε* could be adopted in the analysis, potentially further restricting the enumerated candidate set. Indeed, the parameter‐sensitivity analysis in Figure S2a indicates that, within the range of *ε* values sufficiently broad to encompass experimental uncertainty, smaller *ε* tends to yield higher assignment accuracy.

Furthermore, by combining the present framework with automated detection of diffraction spots and layer lines, for example, using deep learning, the current semiautomated workflow could potentially be extended to fully automated (*n*,*m*) assignment. Such automated feature extraction is expected to further reduce initial measurement errors and manual workload, while improving the efficiency of high‐throughput chirality characterization. Overall, the local candidate search and cross‐check‐based framework presented here already offers high practical utility, while still leaving room for further improvement, particularly for large‐diameter CNTs close to the zigzag structure.

### 4.5 Comparison With Existing NBED‐Based Chirality‐Assignment Methods

Finally, we briefly compare representative existing methods for SWCNT chirality assignment from NBED patterns to clarify the position of the method developed in this study within the current analytical framework. Several established approaches are available for NBED‐based chirality assignment, including methods based on Bessel‐function analysis, methods using multiple diffraction features or simultaneous comparison with simulated diffraction patterns, and recently developed AI‐assisted classification methods (Table 3). The uncertainty‐based candidate search and overlap‐ratio ranking developed in this study combine several practical advantages, including freedom from absolute reciprocal‐space scale calibration, applicability to tilted CNTs, high computational efficiency, and high physical interpretability. At the same time, the manual operation workload, which has been a major practical limitation of conventional NBED analysis, is reduced through implementation of the present method in a semiautomated analysis system.

**TABLE 3:** Qualitative comparison of representative SWCNT chirality‐assignment methods based on NBED patterns.

Method	Absolute reciprocal‐space scale calibration	Applicability to tilted CNTs	Computational efficiency	Manual operation workload	Interpretability	Refs.
Direct Bessel‐order identification	Not Required	Limited	High	Low	High	Liu and Qin [45]
Robust Bessel‐function analysis	Not Required	Limited	High	Medium	High	Jiang et al. [36]
Full‐pattern simulation matching	Required	Supported	Medium	High	High	Meyer et al. [44]
Intrinsic layer‐line spacing	Not Required	Supported	High	Medium	High	Jiang et al. [37]
AI/ViT classification	Not Required	Supported	Inference: High; Training: Low	Very Low	Medium	Iwasaki et al. [50]
Uncertainty‐based candidate search and overlap‐ratio ranking	Not Required	Supported	High	Low	High	This work

Another important challenge in previous studies has been the limited scale of quantitative validation. Among the representative methods summarized in Table 3, with the exception of the AI/ViT classification method reported by our group in 2026, experimental or simulation‐based validation was generally demonstrated using relatively limited sets of CNT chiralities and tilt conditions rather than broad, systematically labeled datasets. In contrast, Ref. [50] established a broad simulation‐based diffraction dataset with known ground‐truth chirality labels, and a defined subset of this dataset was used for validation in the present study [51]. The present validation used 2394 experiment‐mimicking diffraction patterns corresponding to 266 chiralities, spanning diameters of 0.48–2.3 nm, chiral angles of 0°–30°, and tilt angles of 0.5°–16.5° in 2° increments. Ref. [50] employed 5586 simulated test patterns covering the same 266 chiralities over a broader tilt‐angle range. The full labeled dataset, including the base simulated diffraction patterns and experiment‐mimicking images, is publicly available in Zenodo [51].

The availability of such broad, labeled diffraction datasets provides a useful basis for further development of physically interpretable and increasingly accurate chirality‐assignment methods. In turn, these physics‐based frameworks and datasets may support further improvement of AI classifiers aimed at minimizing human workload, while also extending the applicable range toward more challenging conditions, including larger CNT tilt angles, large‐diameter chiralities, multi‐walled CNTs, and CNT bundles.

## 5 Conclusion

In this study, we developed a chirality‐assignment protocol for NBED analysis of SWCNTs by integrating local candidate search under assumed manual measurement errors and CNT tilt with cross‐check‐based candidate filtering and ranking. Validation using 2394 simulated diffraction patterns demonstrated that the assignment accuracy improved from 71.6% for conventional TRUNC‐based integer conversion to 97.7% with the proposed protocol. The method also reduced bias in dataset‐level structural statistics, including mean CNT diameter, mean chiral angle, and metallic CNT fraction. In addition, semiautomated feature extraction and candidate screening improved practical usability by reducing analysis time, enhancing reproducibility, and minimizing operator dependence.

Analysis of the individual correction components showed that the overlap ratio introduced in this study plays the central role in final candidate ranking, whereas tilt‐parameter consistency serves as a complementary metric for verifying the physical plausibility of candidates. This indicates that the improvement arises not from exhaustive exploration of the full (*n*,*m*) space, but from stable discrimination among competing candidates generated within the uncertainty‐defined ranges derived from the measured diffraction spacings.

Residual misclassifications after correction were mainly observed for large‐diameter, low‐chiral‐angle CNTs, where neighboring chiralities become intrinsically difficult to distinguish because of reduced separability in diffraction geometry. Thus, although the proposed method is highly effective under realistic manual‐analysis conditions, it does not completely eliminate structurally difficult cases. Overall, this study provides a physics‐based, accurate, reproducible, and practical (*n*,*m*) assignment protocol for SWCNT electron diffraction analysis, and it offers a useful foundation for future high‐throughput chirality analysis through integration with equatorial‐line intensity information, automated feature extraction, and AI/MI‐based approaches.

## Conflicts of Interest

The authors declare no conflicts of interest.

## Supporting information




**Supporting File 1**: smtd71039‐sup‐0001‐SuppMat.docx.

**Supporting File 2**: smtd71039‐sup‐0002‐SuppMat.zip.

## Data Availability

The simulated diffraction dataset that supports the findings of this study is openly available in Zenodo at https://doi.org/10.5281/zenodo.22084504 [51]. Supplementary figures and tables, the analysis tools used in this study, and a detailed user guide describing the analysis workflow and operation procedures are provided in the Supporting Information.
