# Supplementary material for: Accurate (n,m) Assignment of Single‐Walled Carbon Nanotubes From Electron Diffraction Patterns Via Local Candidate Search and Self‐Consistency Correction
Source: Small Methods. 2026 Sep 16;10(20):e71039. doi: 10.1002/smtd.71039 (PMC13706644; doi:10.1002/smtd.71039)
Supplement: Supplementary file 1 [file SMTD-10-e71039-s001.docx]

**Supporting Information**

**Accurate (n,m) Assignment of Single-Walled Carbon Nanotubes from Electron Diffraction Patterns via Local Candidate Search and Self-Consistency Correction**

Hirotaka Inoue^1,2*^, Hua Jiang^1^, Rui Iwasaki^2^, Anastasios Karakassides^1^, Toshihiko Fujimori^2,3^, Esko I. Kauppinen^1*^

^1^Department of Applied Physics, School of Science, Aalto University, Puumiehenkuja 2, 02150 Espoo, Finland.

^2^Sumitomo Electric Industries, Ltd., 1-1-3 Shimaya, Konohana-ku, Osaka 554-0024, Japan.

^3^Institute of Applied Physics, University of Tsukuba, Tsukuba, 305-8573, Japan

^*^Corresponding author:

Hirotaka Inoue ([inoue-hirotaka@sei.co.jp](mailto:inoue-hirotaka@sei.co.jp))

Esko I. Kauppinen ([esko.kauppinen@aalto.fi](mailto:esko.kauppinen@aalto.fi))

**Figure S1.** (a) Before and (b) after band-pass filtering of an experimental diffraction pattern of a SWCNT.

**Table S1.** Predefined uncertainty windows and filtering parameters used for local candidate search in (*n*,*m*) assignment.

Note: The range from cos*τ*_min_ to cos*τ*_max_ is used as a tolerance window for the calculated cos*τ* values, including measurement uncertainty; cos*τ*_max_ > 1 does not represent a physically possible tilt angle. The error windows for *δ*, *d*_2_, and *d*_3_ are defined relative to the measured values *δ*′, *d*_2_′, and *d*_3_′.

**Figure S2.** Parameter-sensitivity analysis of the (*n*,*m*)-assignment protocol. Fractions of assignment outcomes for 2394 simulated test patterns as a function of (a) the relative-error parameter *ε*, (b) the lower bound of the calculated-cos*τ* tolerance window, cos*τ*_min_, and (c) the threshold of |cos*τ*_3_ − cos*τ*_2_|. Here, *ε* defines Δ*δ* = ±*εδ*′ and Δ*d*_2_ = ±0.5*εd*_2_′, Δ*d*_3_ = ±0.5*εd*_3_′.

**Figure S3.** Evaluation of (*n*,*m*) assignment using only the tilt-parameter consistency criterion, defined by minimization of |cos*τ*_3_ - cos*τ*_2_|. (a) Color-coded assignment accuracy plotted on the SWCNT (*n*,*m*) chirality map. Each hexagon represents a specific (*n*,*m*) species, and the color indicates the fraction of correctly assigned diffraction patterns for that species. (b) Distribution of assignment errors in *n* and *m*, expressed as Δ*n* and Δ*m*. (c,d) Dependence of the mean assignment error, defined as the bin-averaged value of |Δ*n*| + |Δ*m*|, on (c) CNT diameter and (d) chiral angle.

**Figure S4.** Evaluation of (*n*,*m*) assignment using only overlap-ratio maximization for final candidate ranking. (a) Color-coded assignment accuracy plotted on the SWCNT (*n*,*m*) chirality map. Each hexagon represents a specific (*n*,*m*) species, and the color indicates the fraction of correctly assigned diffraction patterns for that species. (b) Distribution of assignment errors in *n* and *m*, expressed as Δ*n* and Δ*m*. (c,d) Dependence of the mean assignment error, defined as the bin-averaged value of |Δ*n*| + |Δ*m*|, on (c) CNT diameter and (d) chiral angle.

**Table S2.** Comparison of experimental agreement values reported in Ref. [50] and obtained in the present study under different AI confidence-score thresholds and manual assignment procedures.

**Figure S5.** Detailed analysis of the experimental NBED pattern showing the largest discrepancy between the AI prediction and manual assignment. (a) Experimental NBED pattern. Yellow arrows indicate the pair of diffraction spots associated with L_3_ that approach each other near the meridian. (b) Chirality, CNT diameter, and chiral angle obtained from the AI prediction and manual assignments without and with cross-check correction. (c–g) Simulated diffraction patterns of the manually assigned (19,10) CNT at tilt angles of 0°, 5°, 10°, 15°, and 20°, respectively, without added image noise, beam-stop shadows, or other perturbations. Yellow arrows indicate the corresponding L_3_ diffraction spots. (h) Distance between the indicated L_3_ spots as a function of CNT tilt angle for the simulated (19,10) CNT.
