# Supplementary material for: Accurate (n,m) Assignment of Single‐Walled Carbon Nanotubes From Electron Diffraction Patterns Via Local Candidate Search and Self‐Consistency Correction
Source: Small Methods. 2026 Sep 16;10(20):e71039. doi: 10.1002/smtd.71039 (PMC13706644; doi:10.1002/smtd.71039)
Supplement: Supplementary file 2 [file SMTD-10-e71039-s002.zip › SWCNT Chirality-Assignment Tools/UserGuide.pdf]

User Guide for
SWCNT Chirality-Assignment Tools

Overview

This document provides instructions for using the semi-automated analysis tools developed for assigning the chirality indices (n,m) of single-walled carbon nanotubes (SWCNTs) from nanobeam electron diffraction (NBED) patterns. The analysis workflow consists of the following two components:

I. An ImageJ-based procedure for extracting diffraction-pattern features and measurement parameters.

II. An Excel VBA macro for uncertainty-based candidate generation, self-consistency screening, overlap-ratio-based ranking, and final (n,m) assignment.

File List

The following files are provided in the Supporting Information:

- **SWCNT_NBED_Analysis.xlsm**
  Excel workbook containing the VBA macros and calculation sheets.
- **sample images** (folder)
  Representative NBED patterns.
- **UserGuide.pdf**
  This instruction document.

How to Use

1 ImageJ Installation

1.1 Download ImageJ from the URL below.

download URL: https://imagej.net/ij/download.html

*Fiji (https://imagej.net/software/fiji/downloads) is also acceptable.

1.2 Unzip the downloaded file and move the “ImageJ” folder to a local, such as C:¥Program Files.

2 Excel Macro Setup

2.1 Open the attached file “SWCNT_NBED_Analysis.xlsm”.

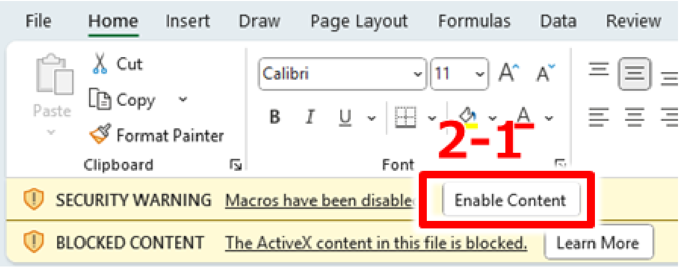

2.2 Enable macros and active content only for the supplied workbook. If macros are still not enabled, change the file security settings following the steps (2.3, 2.4).

2.3 In File Explorer, right-click the Excel file → Properties → General tab → check “Unblock” under Security → OK.

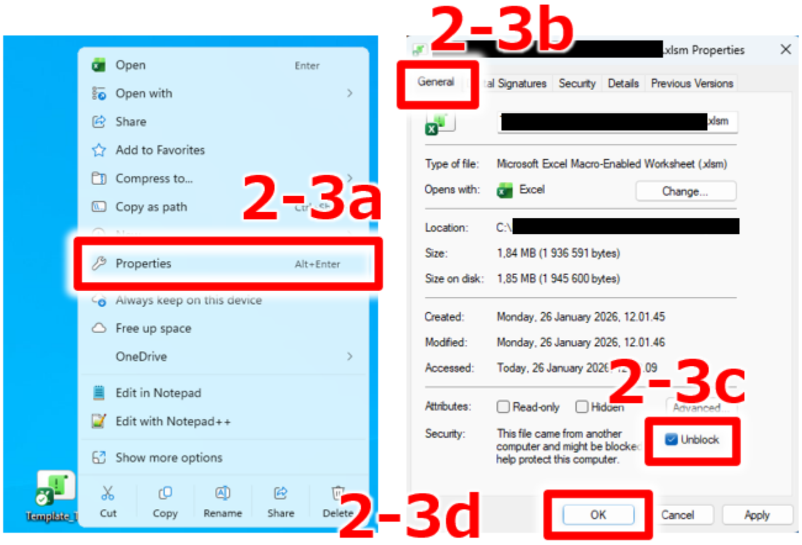

2.4 (option) Only if the ActiveX controls in this workbook remain disabled: Open the Excel file and go to File → Options → Trust Center → Trust Center Settings → ActiveX Settings. Select "Enable all controls…" and click OK.

* Restore the original ActiveX settings after completing the analysis.

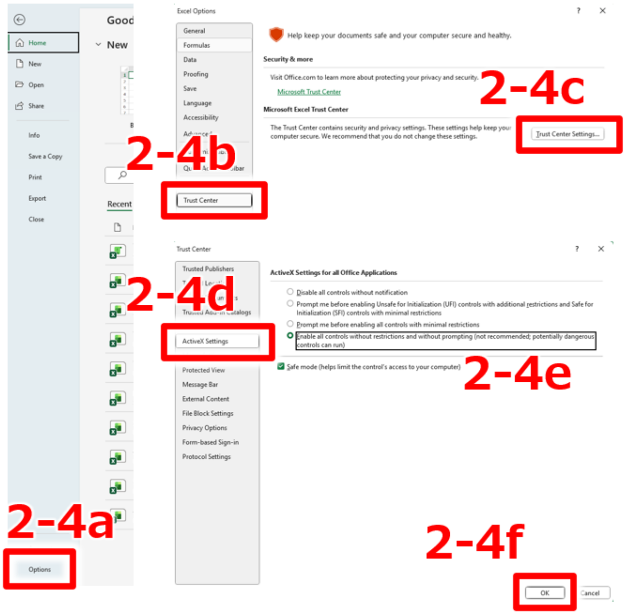

2.5 Select the "Calc-0" sheet.

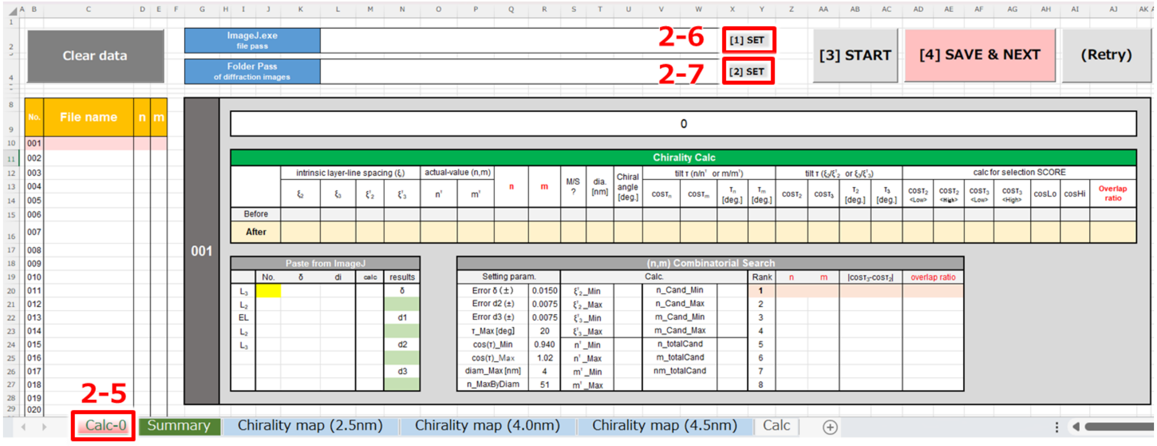

2.6 Click "[1] SET" (cell X2) and specify the file path to ImageJ.exe.

2.7 Click "[2] SET" (cell X4) and specify the folder path containing the diffraction images to analyze. All .dm3/.png/.jpg/.tif files in the folder will be listed in column C.

3 Diffraction Image Analysis

3.1 Click “[3] START” (cell AA2) to start the main analysis.

3.2 After the image opens in ImageJ, use the Line Selection tool (yellow line) to select the equatorial line (EL) of the diffraction pattern.

3.3 Note: If the line is hard to see, adjust the contrast using the MINIMUM and MAXIMUM sliders in the B&C window before selecting the EL.

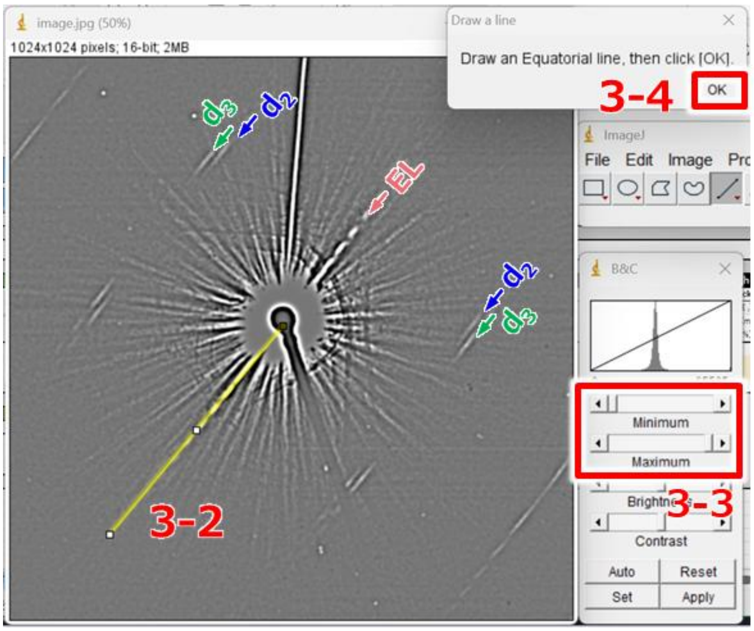

3.4 Click “OK”.

3.5 In the Plot Profile window, click 3–7 successive extrema to mark their positions. (yellow points).

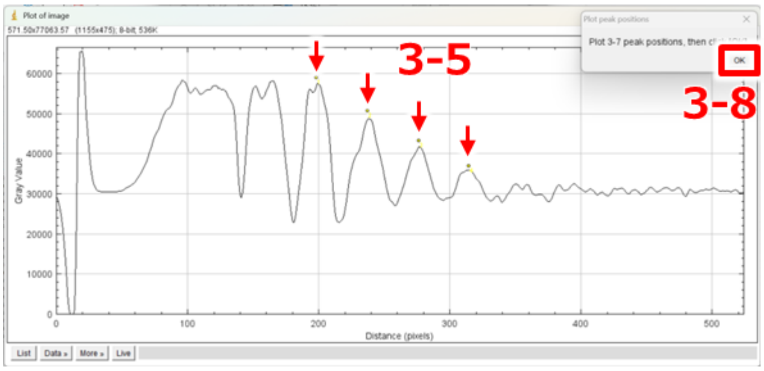

3.6 Note: Select either maxima or minima consistently, and proceed from one side to the other without skipping any extrema. The measured equatorial-line spacing δ' is calculated as the median of the spacings between adjacent selected extrema.

3.7 Note: If you want to clear all yellow points in the Plot Profile window, slightly resize the window with your mouse to reset the point selection.

3.8 Click “OK”.

3.9 In the Plot Profile window, select the five peaks in this order: d_3, d_2, EL, d_2, d_3 (yellow points).

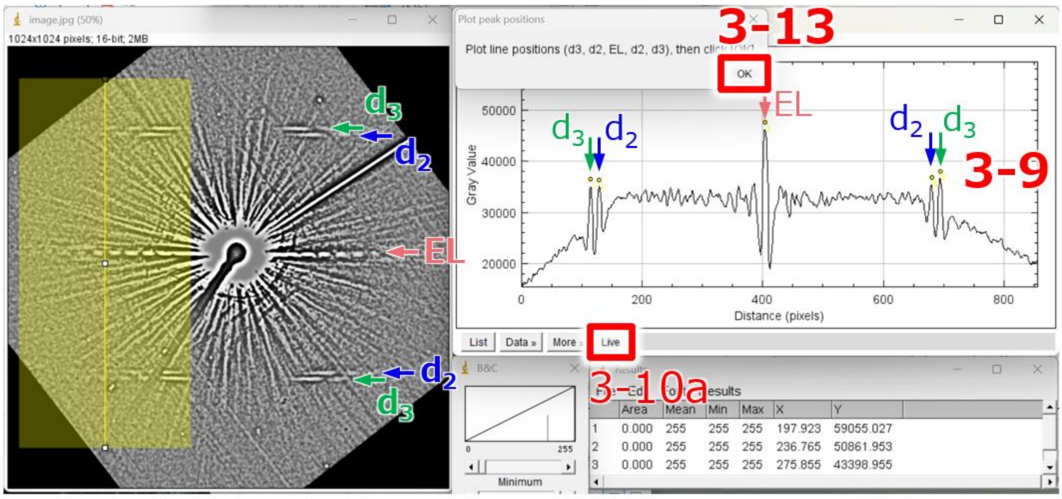

3.10 Note: If some peaks are unclear with the initial line selection, click “Live” in the Plot Profile window, then return to the diffraction image and adjust the yellow line position/length. If you accidentally delete the initial line, select “Straight Line” in the main ImageJ window and draw the line again on the diffraction image. Then select the “Multi-point Tool” and perform peak selection in the Plot Profile window.

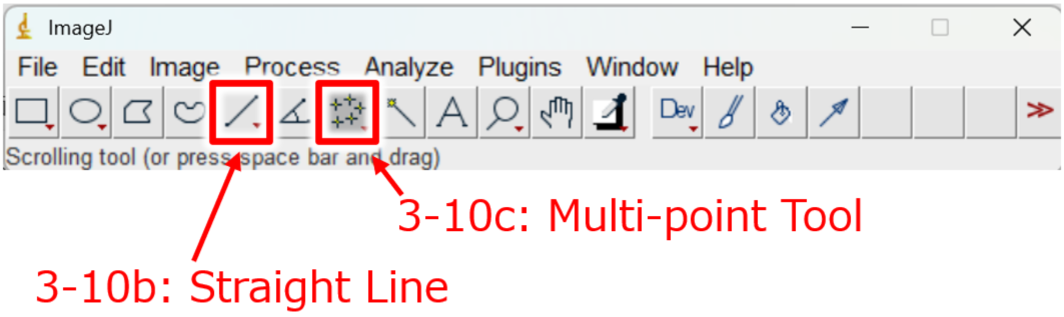

3.11 Note: Start from either the right or left, but always select adjacent peaks in order.

3.12 Note: For armchair CNTs, d_3 and d_2 overlap, so click two points at the same X position (slightly shifted in the Y direction).

3.13 Click “OK”.

3.14 If the ImageJ analysis was completed correctly, click “OK” to import the results into Excel. Otherwise, click “Cancel” to discard the current measurements.

3.15 Review the assigned (n,m) indices. If the result is acceptable, click “[4] SAVE & NEXT” (cell AD2) to save the result to the “Calc” sheet and proceed to the next image.

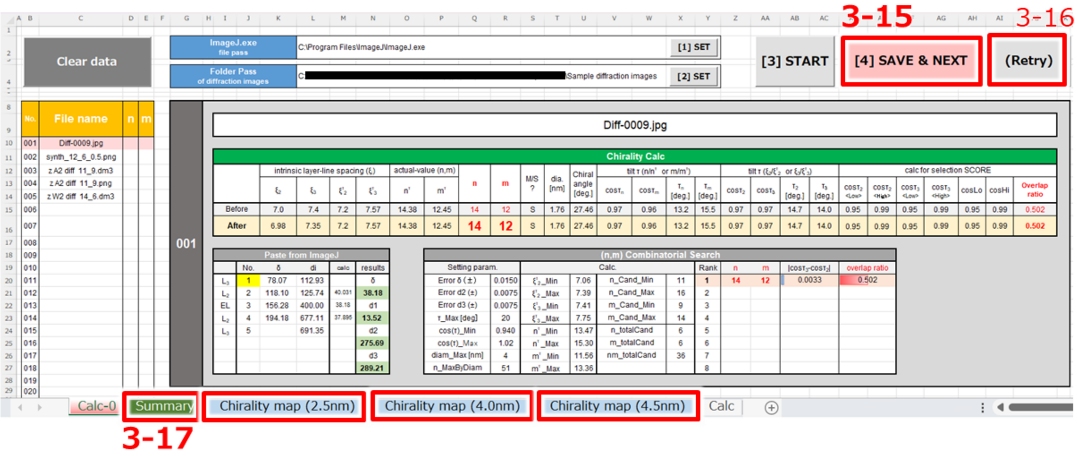

3.16 Note: If you want to rerun the analysis, click “(Retry)” (cell AJ2).

3.17 After analyzing all diffraction images, you can review the results in the “Summary” and “Chirality map” sheets.

Test Environment

The tools were developed and tested using the following software:

- OS: Windows 11
- Excel: Microsoft Excel LTSC, Version 2408 (64-bit)
- ImageJ: 1.53k

Contact Information

If you encounter any problems or have questions regarding the analysis tools, please feel free to contact us at the following email addresses:

Dr. Hirotaka Inoue

- hirotaka.inoue.01@gmail.com
